# The 5 A’s of firearm safety counseling: Validating a clinical counseling methodology for firearms in a simulation-based randomized controlled trial

**DOI:** 10.1016/j.pmedr.2022.101811

**Published:** 2022-05-05

**Authors:** Katherine Hoops, Alexander McCourt, Cassandra K. Crifasi

**Affiliations:** a Johns Hopkins University School of Medicine, Department of Anesthesiology and Critical Care Medicine, United States; b Johns Hopkins Bloomberg School of Public Health, Department of Health Policy and Management, United States

**Keywords:** Firearm, Violence prevention, Injury prevention, Medical education, Simulation

## Abstract

The 5 A’s of Firearm Safety Counseling is a novel framework by which clinicians can approach firearm injury prevention counseling. To evaluate this methodology as a tool for clinicians, a single-center, simulation-based randomized controlled trial was performed with clinical trainees in psychiatry, medicine, and pediatrics in an urban quaternary care center. Participants received didactic education on firearm injury epidemiology and evidence-based policies and training on a specific counseling framework, the 5 A’s of Firearm Safety Counseling which they then implemented in a simulation setting with standardized patients. Of the 29 participants who were randomized, 28 completed the trial. Most participants were psychiatry trainees (residents or subspecialty fellows). While over 60% of participants were uncomfortable or extremely uncomfortable counseling on firearm injury prior to the interventions, only 4% reported being uncomfortable after receiving education and participating in simulated encounters. There was no significant difference between the quality and content of the counseling provided before and after the didactic-only session. There was a significant difference between the quality and content of the counseling provided before and after the specific training on the 5 A’s for Firearm Safety Counseling strategy. The 5 A’s for Firearm Safety Counseling is a promising educational tool to improve quality, content, and comfort delivering patient-centered counseling on firearm injury prevention in a simulation-based setting. These findings suggest that further validation in a clinical setting is warranted given there is an urgent need for feasible and effective firearm injury prevention strategies among clinicians.

## Highlights

• Comfort providing firearm safety counseling substantially increased after education.
• Quality and content of counseling improved with training on the 5 A’s algorithm.
• The 5 A’s is an effective tool to improve firearm counseling in a simulation setting.

## 1 Introduction

Four in ten adults in the United States live in a home with a gun, and one in three children live in a home with a gun (Van Green, 2021, Schuster et al., 2000). Meanwhile, a large body of evidence demonstrates that the presence of firearms in the home is associated with increased likelihood of suicide (Wintemute et al., 1999, Kellermann et al., 1992, Miller et al., 2007), homicide (Cummings et al., 1997, Wiebe, 2003, Dahlberg et al., 2004, Kellermann et al., 1993), and unintentional injuries (Miller et al., 2001). A nationally representative survey of U.S. firearm owners found that fewer than 20% of respondents rated physicians as effective messengers about safe storage practices (Crifasi et al., 2018). Clinicians can be effective at motivating patients and families to adopt safe storage practices (Grossman et al., 1995, Rowhani-Rahbar et al., 2016); however, they need training and a framework to improve their knowledge and self-efficacy in providing this type of counseling (Betz and Wintemute, 2015). In surveys seeking to characterize diverse groups of medical trainees’ barriers to counseling on firearms, residents cited lack of time in visits and lack of education on firearms, safety devices, and counseling methods as some of the main impediments to providing firearm safety counseling (Hoops and Crifasi, 2019, Pallin et al., 2022). In order to address those barriers, one of the most frequently requested forms of education was on “specific language to use when talking to families.” (Hoops and Crifasi, 2019) Taken together, these findings highlight the need for additional research to explore how to better prepare physicians to provide high quality anticipatory guidance on safe gun storage.

Providing guidance on a range of injury prevention topics is a core clinical competency for all health care providers. However, prior research has found that most clinicians never or rarely provide firearm safety counseling to their patients (Grossman et al., 1995, Hoops and Crifasi, 2019, Price et al., 2013, Ketterer et al., 2020, Butkus and Weissman, 2014). Validated models exist for counseling on certain behaviors and conditions associated with major health risk reduction, including smoking cessation and weight loss, that could serve as a model for improving the delivery of firearm safety counseling. Implementation of the 5A’s model (Ask, Advise, Assess, Assist, Arrange) has been proven effective at addressing complex health behaviors: it has been shown to improve motivation to quit and increase quit attempts among smokers (Quinn et al., 2009, [21]) as well as increasing motivation to lose weight, change diet behaviors, and actual weight loss among obese patients (Welzel et al., 2018, Alexander et al., 2011, Searight, 2018). The use of validated counseling algorithms by clinicians has been shown to improve rates of counseling, effectiveness of counseling, and, consequently, patient outcomes (Ha and Longnecker, 2010).

Simulation is a valuable tool used in many aspects of medical education, including that focusing on high-stakes, complex communication (Evans and Taubert, 2019, Weller, 2004, McGaghie et al., 2010, Henner and Boss, 2017). The use of education including role-play has already been shown to be effective at sustainably improving attitudes and practices regarding firearm safety counseling (McKay et al., 2020). Simulation including standardized patient actors adds fidelity to peer role-play scenarios (Gelis et al., 2020). Our objective was to evaluate a resource for clinicians that provided a specific counseling strategy and educational program which could feasibly be implemented in existing medical education curricula. This counseling methodology stands to improve physician counseling on firearms across a range of disciplines and, thereby, prevent gun injuries. We hypothesized that physicians would improve the quality and content of their counseling in the observed simulation setting after receiving training on the 5A’s model compared to their baseline.

## 2 Methods

To determine whether the implementation of this framework was possible in a simulation setting and to assess its effect on the quality and content of counseling provided by clinicians, we designed a single-blind, randomized controlled trial utilizing simulated patient encounters in our institution’s medical simulation center. We worked with experts in simulation-based education and standardized patient education and assessment to refine study logistics and employ and train standardized patients. Based on prior work demonstrating that residents rarely received didactic education on firearm injury, were unfamiliar with safe storage devices, and were interested in learning specific frameworks for providing counseling, our research team and simulation-based education experts designed an educational intervention and counseling strategy that met their needs (Hoops and Crifasi, 2019). This study was reviewed and approved by our Institutional Review Board.

### 2.1 Participants

Learners were recruited through their enrollment in residency or fellowship training programs at our institution, which is a large, quaternary care center in an urban setting. Any physician (trainee or faculty) was eligible to participate. We convened three groups of 5–15 learners (a total of 29 learners) from each of four training programs: psychiatry residency, pediatric psychiatry fellowship, internal medicine residency, and internal medicine-pediatrics residency. Sample size was determined based on enrollment in these training programs and trainees’ availability for half-day educational programming balanced with their clinical demands.

Upon arrival to the study site (our institution’s simulation center), learners provided informed consent including permission to audio and video record all simulated encounters and completed a preliminary survey to characterize confidence providing counseling on a range of safety-related topics as well as gather demographic information. One participant declined to participate in the study but did want to receive education; that person was, therefore, exempt from the surveys and was not video or audio recorded but was allowed to participate in the educational sessions and simulated encounters. Next, prior to any further discussion or education, all learners were observed in a fifteen-minute simulated encounter (baseline scenario) with a standardized patient (a young, recently returned veteran with symptoms of post-traumatic stress disorder) with triggers to assess for the presence of firearms in the home.

After completion of the baseline scenario, we randomized learners to two groups: a control and intervention group.

### 2.2 Description of the educational intervention

We created a brief (thirty-minute) lecture on the epidemiology of firearm deaths in the United States including homicide, suicide, and unintentional injuries. We included data on differences by age, gender, and race. The lecture also included information on key firearm policies such as extreme risk protection orders, child access prevention laws, and voluntary transfers as well as research on the impacts of gun storage practices on injury risk. Finally, the lecture covered specific gun storage methods, provided opportunity for physicians to handle common storage devices such as cable locks, trigger locks, and small gun safes with digital keycode locks, and allowed time for questions and discussion.

**Fig. 1:**
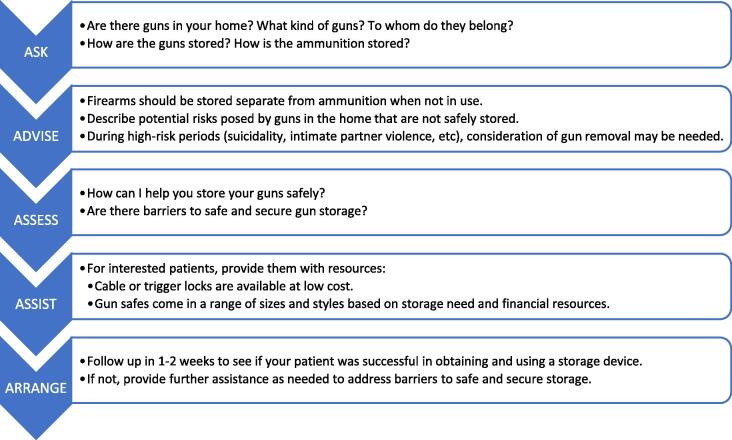
The 5 A’s Counseling Algorithm Applied to Firearm Safety Counseling.

We also created a counseling algorithm specific to firearms (5 A’s of Firearm Safety Counseling) based on the literature supporting the use of the 5 A’s model as a motivational interviewing and brief intervention framework, diagrammed in Fig. 1. (Crifasi and Hoops, 2019) In brief, using this model, clinicians *ask* the patient about the presence of guns in the home, and, if guns are present, characterize the types of guns and the current storage method. Of particular importance, the clinician must *advise* the patient in clear and personalized language that the presence of firearms in the home is associated with increased likelihood of suicide (Wintemute et al., 1999), homicide (Cummings et al., 1997), and unintentional injuries (Miller et al., 2001). Safe storage practices (i.e., keeping all firearms stored unloaded, locked, separate from ammunition and/or with extrinsic safety devices) are protective against unintentional injuries and suicide (Grossman et al., 2005). Clinicians should a*ssess* the patient’s interest in implementing safe storage practices or removing guns from the home. Then they *assist* the patient in accessing resources for safe storage by, for example, counseling them on the availability of gun safes at local and online retailers as well as lower cost options such as trigger locks and cable locks. Finally, clinicians a*rrange* follow up with the patient to ensure adoption of the safe storage practices or other firearm safety behavior changes.

### 2.3 Testing of the educational intervention and counseling framework

**Fig. 2:**
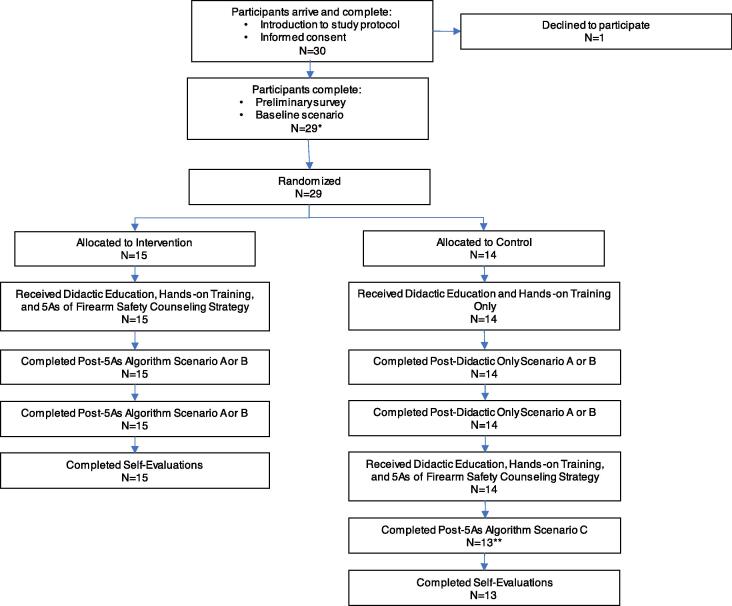
Flow Diagram of Participant Randomization and Study Activities. *One participant arrived late and did complete the baseline survey but did not complete a baseline scenario prior to receiving education as part of the intervention group. **One participant withdrew prior to the end of the session and was unable to complete the final scenario or self-evaluation.

After randomization, the *control* group received the thirty-minute lecture on basic firearm injury epidemiology and the importance of firearm safety counseling as well as hands on training with gun safes and external locking devices, and the *intervention* group received a forty-five-minute lecture with the same content as the first group but with the addition of specific education on the 5 A’s of Firearm Safety Counseling strategy. After their educational sessions, all study participants returned to the standardized patient interactions to participate in two scenarios (post-education scenarios) with standardized patients in which they encounter (A) a young person with depression and attention deficit hyperactivity disorder and (B) a pregnant young woman with repeated injuries due to intimate partner violence with triggers to assess for the presence of firearms in the home. They were given fifteen minutes to complete these focused encounters. Following the two post-education scenarios (either post-5 A’s algorithm or post-didactic only), the *control* group (didactic only) received an additional fifteen-minute didactic on the 5 A’s of Firearm Safety Counseling strategy and performed an additional simulated encounter with an additional standardized patient (an elderly veteran with depression) to ensure educational equity among all participants in the study. Refer to Fig. 2 for a study flow diagram (Schulz et al., 2010).

After completing all assigned scenarios, participants completed self-evaluations that examined their assessments of the quality of the education received and its usefulness and effectiveness.

## 3 Data abstraction

In order to systematically abstract data on counseling content and quality, we created a scoring rubric based on essential elements of risk-based, patient-centered firearm safety counseling. Because the use of simulated patient encounters for clinician training on firearm safe storage counseling was entirely novel in the current literature, no previously validated rubric was available for use in this project. The rubric included whether the provider asked about the presence of guns in the home, provided patient-specific counseling on risks of guns in the home and safe storage options, or made a plan to follow up with the patient; there were a total of thirteen subelements to assess each of these domains. For each subelement of the rubric, participants were rated Addressed (2 points), Partially Addressed (1 points), Not Addressed (0 point), or Not Applicable. Not applicable was reserved for a few circumstances where a Standardized Patient provided incorrect information based on the standardized prompts for character development that impacted the questions asked by the participant or counseling provided. For elements marked as Not Applicable, the points possible for that element were removed from the denominator. Participants who scored fewer than 10 points for a given simulation were marked as Insufficient, 11–14 points was Approaching Sufficient, 15–18 points was Sufficient, 19–22 points was Approaching Proficient, and 23–26 points was Proficient.

The video review order, including baseline simulations, was randomized to reduce reviewer bias when scoring each simulation. The reviewers (K.H. and C.C.) watched each video simultaneously using virtual screen sharing and independently scored each simulated encounter using the rubric. The reviewers then immediately debriefed on each scenario to discuss any differences in scoring. When necessary, sections of videos were re-reviewed to resolve any remaining scoring differences so that the reviewers agreed on a final score. Preliminary validation of the review process demonstrated 52% agreement in the first twenty-five recordings reviewed but 100% agreement in the latter third of that sample indicating a reassuring degree of inter-rater reliability with application of the rubric.

## 4 Analytic methods

Once data abstraction was complete, we compiled a dataset that contained a numerical score for each simulation, an indicator for which scenario was associated with a given score, and an indicator for whether a score was from the baseline encounter, post-didactic-only, or post-5 A’s algorithm. We used paired t-tests to assess for differences across learner groups comparing the baseline to didactic-only, baseline to post-5 A’s, and didactic-only to post-5 A’s. We also assess for differences between learning groups for each of the domains of the 5 A’s (available as supplemental materials). Our analytic sample with relevant sample sizes is as follows: (1) “baseline” represents each participant’s control or pre-education encounter, excluding one participant who arrived late (N = 28); (2) “post didactic-only” represents the encounters performed by the 14 individuals who were randomized to receive the didactic only after which they performed two encounters (N = 28); (3) “post-5As” represents all encounters performed following education on the 5As framework with the intervention group (15 participants) performing two encounters after education and the control group (13 participants given one withdrew early as shown in Fig. 2) performing one encounter after a supplemental lecture on the framework (N = 43, intervention group n = 30, post-educational-equity control group n = 13).

Results are presented as differences in mean scores with significance set at p less than 0.05. Analyses were conducted in Stata 17 (StataCorp. Stata statistical software: release 14. College Station, TX: StataCorp LP, 2015).

## 5 Results

Twenty-nine trainees participated in the simulation-based RCT. Trainee programs and program year are provided in Table 1. Nearly half were Psychiatry residents and most (58%) were in their second or third program year.

**Table 1:** Participant Programs and Program Year in the 5 A’s for Firearm Safety Counseling Randomized Controlled Trial (N = 29).

	n (%)
**Program**	
Psychiatry Residency	14 (48)
Child and Adolescent Psychiatry Fellowship	5 (17)
Internal Medicine Residency	3 (11)
Internal Medicine/Pediatrics Residency	7 (24)
**Program Year (Post Graduate Year)**	
1	1 (3)
2	10 (34)
3	7 (24)
4	6 (21)
5	–
6	5 (17)

The mean baseline score for all learners was 14.8 (approaching sufficient) (Table 2). The sample size was not adequate to assess differences between clinical disciplines. After randomization, those who received the didactic-only training had a slightly higher mean score (15.8, sufficient); however, this was not statistically significantly different from the baseline (p = 0.45). There was a significant increase in the mean score for those post-5 A’s (18, sufficient) compared to both baseline (p = 0.003) and post-didactic-only (p = 0.03). Comparing within learners (i.e. individuals’ pre/post assessments), findings were consistent with significant improvements in counseling scores post-5 A’s (results not shown). When comparing across the domains of the 5 A’s, mean score improvements were noted comparing baseline to post-5A’s for Advise (e.g., providing patient-specific risk counseling), Assess (e.g., assessing whether the patient was ready to consider changing their gun storage behaviors), and Arrange (e.g., setting up a time to follow up with the patient on safe storage behaviors) (supplemental Table 1).

**Table 2:** Results of T-tests Assessing Differences in Means between Learner Groups in the 5 A’s for Firearm Safety Counseling Randomized Controlled Trial (N = 29).

Learner Group	Mean	SD	t	p-value
Baseline	14.8	4.7	−0.75	0.45
Post Didactic-Only	15.7	4.6		
Baseline	14.8	4.7	−3.08	**0.003**
Post 5 A’s	18.0	3.9		
Post Didactic-Only	15.7	4.6	−2.24	**0.03**
Post 5 A’s	18.0	3.9		

Participants’ self-assessments also indicate that education was beneficial (Table 3, Table 4). Only 18% of participants reported having received prior didactic education on firearm injury in their training. While less than 40% of participants were comfortable counseling on firearm injury prior to the interventions, 96% endorsed being comfortable after receiving education and participating in simulated encounters. When rating how each aspect of the intervention (didactic education, simulated encounters, 5 A’s counseling framework) affected their comfort counseling, nearly 60% stated that the 5 A’s framework “much improved” their comfort providing counseling, and 50% stated that the didactic session “much improved” their comfort providing firearm-related counseling. All participants felt that the didactic and the framework improved their comfort counseling and 96% felt that the simulated encounters improved comfort providing counseling.

**Table 3:** Participant Initial Survey Results.

Question	Response	n (%) (N = 28)
How comfortable do you feel counseling on injury risk reduction?		
	Extremely comfortable	0
	Somewhat comfortable	7 (25%)
	Neither comfortable nor uncomfortable	7 (25%)
	Somewhat uncomfortable	13 (46%)
	Extremely uncomfortable	1 (4%)
How comfortable do you feel counseling on firearm injury?		
	Extremely comfortable	0
	Somewhat comfortable	4 (14%)
	Neither comfortable nor uncomfortable	7 (25%)
	Somewhat uncomfortable	14 (50%)
	Extremely uncomfortable	3 (11%)
Have you previously participated in simulated patient encounters?		
	Yes, more than 3 times	20 (72%)
	Yes, 1–3 times	4 (14%)
	No	4 (14%)
Have you ever received didactic education on firearm injury?		
	Yes	5 (18%)
	No	19 (68%)
	Do not recall	4 (14%)
How often do you counsel patients on any aspect of firearm safety?		
	Never	4 (14%)
	1–5% of visits	12 (43%)
	6–25% of visits	8 (29%)
	26–50% of visits	0
	51–75% of visits	2 (7%)
	More than 75% of visits	2 (7%)

**Table 4:** Participant Post-Intervention Survey Results.

Question	Response	n (%) (N = 28)
After this session, how comfortable do you feel counseling on injury risk reduction?		
	Extremely comfortable	5 (18%)
	Somewhat comfortable	22 (78%)
	Neither comfortable nor uncomfortable	0
	Somewhat uncomfortable	1 (4%)
	Extremely uncomfortable	0
After this session, how comfortable do you feel counseling on firearm injury?		
	Extremely comfortable	3 (10%)
	Somewhat comfortable	23 (82%)
	Neither comfortable nor uncomfortable	1 (4%)
	Somewhat uncomfortable	1 (4%)
	Extremely uncomfortable	0
After this session, how knowledgeable do you feel about firearms?		
	Extremely knowledgeable	0
	Very knowledgeable	10 (36%)
	Moderately knowledgeable	7 (25%)
	Slightly knowledgeable	10 (36%)
	Not knowledgeable at all	1 (4%)
How did the didactic session today (not including the counseling framework or the simulated patient encounters) affect your comfort with counseling on firearms?		
	Much improved	14 (50%)
	Somewhat improved	8 (29%)
	Slightly improved	6 (21%)
	Not at all improved	0
	I was already very comfortable counseling on firearms	0
How did the simulated patient encounters today (not including the didactic session or the counseling framework) affect your comfort with counseling on firearms?		
	Much improved	10 (36%)
	Somewhat improved	11 (39%)
	Slightly improved	6 (21%)
	Not at all improved	1 (4%)
	I was already very comfortable counseling on firearms	0
How did the 5 A’s framework for firearm injury counseling affect your comfort with counseling on firearms?		
	Much improved	16 (58%)
	Somewhat improved	6 (21%)
	Slightly improved	6 (21%)
	Not at all improved	0
	I was already very comfortable counseling on firearms	0
Would more time for education further improve your comfort with counseling on firearms?		
	Yes	18 (64%)
	Maybe	9 (32%)
	No	1 (4%)
Would a different type of or more education further improve your comfort with counseling on firearms?		
	Yes, more simulated encounters	7 (25%)
	Yes, more didactic lectures on related topics	16 (57%)
	Yes, a webinar or online module	4 (14%)
	Yes, another type of educational session	4 (14%)
	I need no further education on this topic	1 (4%)
Were simulated patient encounters an effective means of learning a new counseling methodology?		
	Yes	19 (68%)
	Maybe	7 (25%)
	No	2 (7%)

## 6 Discussion

The 5 A’s for Firearm Safety Counseling is an effective educational tool to improve quality, content, and comfort delivering patient-centered counseling on firearm injury prevention in a simulation-based setting.

First, affirming our findings from among pediatrics residents (Hoops and Crifasi, 2019) and that of other research groups including one survey of psychiatry residency program directors indicating that 76% of training programs provide no formal training on firearm injury prevention (Price et al., 2010), only 18% of participants could recall having received any didactic training on firearm injury. While there is widespread agreement (Dowd et al., 2012, Talley et al., 2019) that this education is essential for clinicians, only a small portion of clinical trainees are receiving such training, making the dissemination of feasible, effective educational interventions even more urgent.

While, on average, learners scored ‘approaching sufficient’ on their baseline encounters and ‘sufficient’ after didactic-only, scores improved significantly after learning the 5 A’s algorithm. Learning the epidemiology of firearm injury and violence is essential; only with this foundational knowledge can clinicians begin to assess individual patients’ risk for firearm injury (Hoops et al., 2022). Clinicians with this training will be better able to provide focused counseling on access to lethal means for patients who are at risk of suicide or to help patients who are victims of intimate partner violence access policy tools such as domestic violence protection orders when their partner has access to a firearm. However, our findings suggests that while it is helpful to teach trainees basic firearm epidemiology and policy (and this type of education results in improved comfort providing counseling), giving them a tool to navigate a challenging discussion by providing specific questions to pose and an approach to tailored, patient-centered, non-judgmental, risk-based counseling is even more effective. Moreover, the learners uniformly felt more comfortable counseling on firearm injury prevention after the education they received, with nearly 60% feeling that their comfort was “much improved” after the 5 A’s component. Clinicians who are more comfortable providing this counseling are more likely to do so (McKay et al., 2020), and when that counseling also motivates behavior change (Rowhani-Rahbar et al., 2016), it stands to prevent injury and save lives (Violano et al., 2018).

The results of this study should be considered in the context of some limitations. This study had a small sample size; however, the ability to randomize participants to learner groups helps to minimize the likelihood that differences between groups were due to something other than the intervention. Indeed, while we did see some improvements in counseling simply by teaching trainees about basic firearm injury epidemiology, there were clinically (simulated) and statistically significant improvements after the 5 A’s module. This further strengthens our confidence in the effectiveness of our educational tool. However, most of the participants were psychiatry trainees, which may limit the generalizability of the findings to, for example, a primary care setting where the barriers to counseling may be different. The video abstractors did know which scenarios were baseline versus post-invention in this single-blind design so it is possible that bias was introduced in the review and scoring of the recordings, but the review order of the videos was randomized to minimize the likelihood that a trainee’s earlier performance would influence a score. Additionally, the two reviewers simultaneously watched and independently scored the videos. Scores were then immediately discussed, and differences were resolved through discussion and re-review of video segments to ensure appropriate scoring. As the study was conducted in a simulation-based setting, the effectiveness of the 5 A’s for Firearm Safety Counseling may not translate into the clinical setting and the changes seen in clinicians’ counseling may not persist over time. However, prior studies using role-play in a single session did show that the effect of the intervention persisted with improved self-reported rates of firearm safety counseling at six-months post-intervention (McKay et al., 2020).

Future analyses will aim to assess standardized patients’ perception of the quality and content of counseling received as well as their perceptions of rapport-building using the counseling algorithm; given the importance of standardized patients’ feedback (Fanning and Gaba, 2007) (which was not provided in this study), in future iterations of this protocol, particular attention will also be paid to the effect of debriefing with the standardized patient on participants’ self-assessed confidence with counseling and objective assessment of subsequent counseling. Future work will also be devoted to the validation of encounter scoring rubric for use in both simulated and observed clinical encounters with particular attention paid to calculation of inter-rater reliability. Future research should examine the feasibility and effectiveness in inpatient and outpatient clinical settings and to demonstrate durability of the improvements in participants’ counseling with repeated assessments over time. Finally, as this was conducted in one large, urban, training hospital, the results of this study may not be generalizable to other hospital or clinic settings.

## 7 Conclusion

Despite these limitations, this study is the first to our knowledge to test a firearm injury prevention counseling algorithm and educational intervention in a simulation-based setting. The results of this randomized-controlled trial suggest that the 5 A’s for Firearm Safety Counseling is an effective educational tool and counseling framework to improve the quality and content of patient-centered counseling on firearms in a simulation setting. This work also suggests that, when conducted outside the rigor of a randomized trial, this training could be feasibly implemented in and integrated with existing health professionals’ educational programming in an approximately 2-hour session. Work is currently underway to include this training in standard curricula in our institution, but this single session cannot exhaustively address all aspects of a complex and nuanced public health problem with proper attention to all of its attendant issues including racism, policing, community violence, and mental healthcare. As evidenced by the post-intervention average score of “sufficient” rather than “proficient,” more education and training is still needed. This educational program would be best implemented as a part of a complete curriculum addressing health disparities, violence, and injury prevention (Hoops et al., 2022). Nonetheless, this research team is working to translate this simulation-based research into a clinical setting in which clinicians receive education on firearm injury prevention and training on the 5 A’s algorithm (including role play scenarios and viewing of animated simulated encounters to model best practices) before implementing high-quality firearm safety counseling for hospitalized patients. Clinicians urgently need tools at the bedside to reduce patients’ risk of firearm-related injury. Further clinical research assessing the effectiveness of such tools, such as the 5 A’s for Firearm Safety Counseling, while testing implementation strategies to maximize their use for firearm injury prevention is likewise urgent.

## Declaration of Competing Interest

The authors declare that they have no known competing financial interests or personal relationships that could have appeared to influence the work reported in this paper.
